# Self-assessment of surgical ward crisis management using video replay augmented with stress biofeedback

**DOI:** 10.1186/s13037-018-0153-5

**Published:** 2018-04-19

**Authors:** Pasha Normahani, Nita Makwana, Wilhelm von Rosenberg, Sadie Syed, Danilo P. Mandic, Valentin Goverdovsky, Nigel J. Standfield, Usman Jaffer

**Affiliations:** 1 Imperial Vascular Unit, Imperial College NHS Healthcare Trust, London, UK; 2 Department of Postgraduate Medical Education & Simulation, Imperial College Healthcare NHS Trust, St Mary’s Hospital, London, UK; 3 Department of Electrical and Electronic Engineering, Imperial College London, London, UK; 4 Department of Anaesthetics, Imperial College NHS Healthcare Trust, St Mary’s Hospital, London, UK; 5 London Postgraduate School of Surgery, London, UK

**Keywords:** Simulation, Surgery, Self-assessment, Stress, Biofeedback

## Abstract

**Background:**

We aimed to explore the feasibility and attitudes towards using video replay augmented with real time stress quantification for the self-assessment of clinical skills during simulated surgical ward crisis management.

**Methods:**

Twenty two clinicians participated in 3 different simulated ward based scenarios of deteriorating post-operative patients. Continuous ECG recordings were made for all participants to monitor stress levels using heart rate variability (HRV) indices. Video recordings of simulated scenarios augmented with real time stress biofeedback were replayed to participants. They were then asked to self-assess their performance using an objective assessment tool. Participants attitudes were explored using a post study questionnaire.

**Results:**

Using HRV stress indices, we demonstrated higher stress levels in novice participants. Self-assessment scores were significantly higher in more experienced participants. Overall, participants felt that video replay and augmented stress biofeedback were useful in self-assessment.

**Conclusion:**

Self-assessment using an objective self-assessment tool alongside video replay augmented with stress biofeedback is feasible in a simulated setting and well liked by participants.

**Electronic supplementary material:**

The online version of this article (10.1186/s13037-018-0153-5) contains supplementary material, which is available to authorized users.

## Background

Delivery of high quality surgical care is multifaceted, necessitating diligent attention inside and outside of the operating room. High demands are placed on surgical trainees to acquire technical and non-technical skills to perform successful operations but also to manage unwell patients post-operatively.

It is the junior members of the team who often execute the management of post-operative emergencies on the surgical ward. This demands high levels of medical knowledge, clinical skills, leadership, team working and time management skills. Trainees often lack the preparedness to effectively manage such stressful scenarios and this may have a negative impact on patient care [1].

Simulation training provides a safe and structured learning environment allowing for repeated practice and feedback. Simulation based interventions have been shown to positively improve clinical, team-working and patient-physician interaction skills in managing post-operative complications on a simulated surgical ward [2]. Assessment and feedback are integral components of simulation training. Although, this has been traditionally performed by independent assessors, evidence suggests that self-assessment is also reliable [3]. Self-assessment is encouraged in modern surgical education and allows for professional development by prompting the formulation of learning goals [4, 5]. Accurate self-assessment may improve the cost-effectiveness of ward crisis simulation training permitting for more widespread adoption and a sustainable solution to simulation training.

Studies suggest that self-assessment is more accurate when combined with assessment rating scales and retrospective video playback [3]. This form of self-assessment may be well suited to simulated ward crisis as only the trainee will be in a position to evaluate his or her own cognitive process through the scenario. To further enhance self-assessment it may also be beneficial for the trainee to be given further insight into their ‘stress levels’ in the form of biofeedback augmented onto the video playback.

Although a certain degree of stress can facilitate task performance [6], excessive levels can be detrimental. Excessive levels of stress are experienced when demands outweigh the perceived resources to cope [7]. Therefore, being able to highlight times of excessive stress during ward crisis management can identify areas requiring priority attention and may therefore augment-self assessment.

Heart rate variability (HRV) stress indices are detected on ECG and describe variation between consecutive heartbeats. These provide non-invasive quantitative assessment of the autonomic nervous system (ANS) activity on the heart and the balance between parasympathetic and sympathetic systems. The effect of mental stress on HRV has been well documented in the literature [8–12].

The primary aim of this study is to explore the feasibility and trainee attitudes towards using video replay augmented with real time stress quantification for the self-assessment of surgical ward crisis management.

## Methods

### Ward crisis simulation task

Participants were directly approached and recruited from St Mary’s hospital in London, by the authors. Informed consent from participants was sought prior to recruitment. Ethical approval was not required for this training based intervention.

The task was set in the simulation centre, located in the Surgical Innovation Centre, Paterson Building at St Mary’s Hospital. Each room in the simulation center contained all the equipment required for the simulated tasks including a patient bed and clinical adjuncts. Medical student volunteers were briefed regarding the simulation tasks and played the role of patients. The simulated scenarios were further enhanced with props and make up where required. A facilitator was also present in each scenario to provide assistance if required. Each simulated task performance was videoed by an inbuilt camera within the rooms of the simulation centre.

Three simulated ward based scenarios of deteriorating postoperative patients were deployed in separate simulation rooms. Participants were provided with a briefing and then asked to assess and then manage the simulated patient as appropriate. Participants were randomized to the first simulation scenario. The scenarios were designed and written based on real patients and were chosen specifically to simulate common postoperative medical conditions encountered by junior surgical doctors.

Scenario 1 (pulmonary embolism (PE)) centered around a 69-year-old man, 4 day’s post elective total hip replacement surgery who develops sudden onset chest tightness and shortness of breath on a surgical ward. The participants were expected to adequately resuscitate the patient who was in respiratory distress to prevent further deterioration. Printed clinical notes, drug charts, ECG’s, blood gas results and X-rays were available on the participant’s request. Assessment of the notes and drug chart reveal that the patient has a previous history of deep venous thrombosis (DVT) and has missed his last two injections of low molecular weight heparin.

Scenario 2 (lower gastrointestinal bleed) was of a 52-year-old man with a past medical history of diverticular disease complaining of abdominal pain. As the scenario progresses he acutely deteriorates and suffers a significant rectal bleed.

Scenario 3 (sepsis) was focused around a 52-year-old man 7 days post elective right hemicolectomy with a primary anastomosis who is unwell, tachycardic and pyrexial. The patient complains of abdominal pain and has an abdominal drain in-situ with faecal content. If not adequately resuscitated the patient clinically deteriorates during the scenario. Assessment of the ECG also reveals new atrial fibrillation secondary to sepsis.

### Heart rate variability (HRV) assessment

Upon entering the simulation centre continuous ECG recordings were made for all participants using three electrodes placed on the skin, at the lower ribs. The data were sampled at 1 kHz with a custom-made portable device containing a 24 bit analog-to-digital converter (ADC), ADS 1298, the processor MK20DX256VLH7 and stored onto a micro-SD-card. In order to determine the heart rate, the occurrences of R-waves in the ECG were identified applying a previously defined algorithm and software [13], followed by removal of ectopic beats. The time series of RR-Intervals (HRV), the temporal difference between adjacent R-wave occurrences, is the foundation for the HRV assessment.

Six different stress indices were utilised in the time- and in the frequency-domain of the HRV (Table 1).

**Table 1:** Description of heart rate variability (HRV) stress indices

Heart rate variability (HRV) stress indices	Description	Value
Average heart rate (HR)	Average absolute heart rate in time window	Higher: higher stress Lower: lower stress
Sample entropy (SE)	Complexity of the HRV: assumes a complexity loss under constraints (e.g. stress)	Higher: lower stress Lower: higher stress
Low frequency/high frequency ratio (LF/HF)	Balance between sympathetic and parasympathetic nervous systems. LF: associated with sympathetic nervous system, HF: associated with parasympathetic nervous system	Higher: higher stress Lower: lower stress
Standard deviation of RR Intervals (SDRR)	Variation of RR intervals around a mean value	Higher: lower stress Lower: higher stress
Root mean squared of successive differences (RMSSD)	Absolute variation of RR intervals	Higher: lower stress Lower: higher stress
Percentage of RR interval pairs with difference > 50 ms (PRR50)	Compares each pair of adjacent RR intervals and considers the difference between them; if it is larger than 50 ms a counter is increased by one. The ratio of the counter and the number of RR intervals in one time window is pRR50	Higher: lower stress Lower: higher stress

For the calculation of the indices SE and LF/HF, the HRV was resampled with a sampling frequency of 4 Hz. To remove low-frequency heart rate changes that affect the computation of SE, the HRV signal was detrended by subtracting the moving average with a window length of 11 s (for the other five indices, non-detrended data were used). All indices were calculated every 10 s for overlapping time windows with a length of 250 s.

For each participant the whole time series was divided into four parts: the three different scenarios (between entering and leaving the room) and all remaining points in time where the transition periods (250 s) between scenarios and resting were ignored. For each scenario, the 10 time points with the highest stress indices were selected to avoid taking the low-stress parts (for instance the scenario briefing) into account. The means of the 10 values per stress index and scenario were calculated and used for the statistical analysis.

In order to remove subject-specificity of stress indices and to standardise them, the resting values were additionally subtracted from the values during the experiments for additional analysis.

### Development of a self assessment tool

**Fig. 1:**
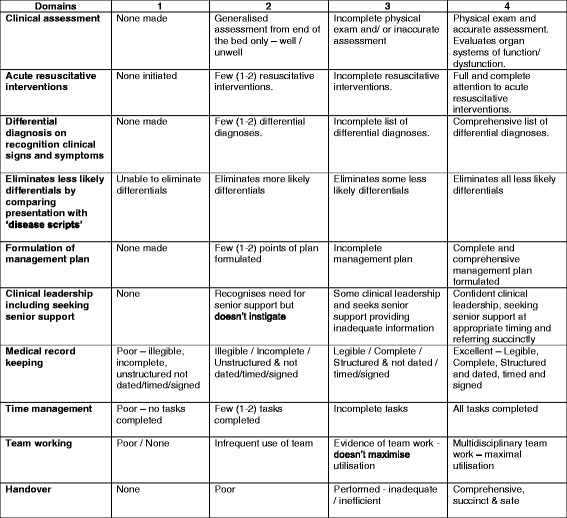
Objective Assessment of Acute Care Skills (OSACS) self-assessment tool

To facilitate self-assessment our team developed the OSACS tool (Objective Structured Assessment of Acute Care Skills). The OSACS tool was developed by two experienced clinicians (U.J and S.S). Content analysis along the principles formulated by Gagné [14] was performed in order to inform the design of OSACS domains. This was further cross referenced with two previously validated assessment tools; the revised Non-Technical Skills (revised NOTECHS) [15] and the Observational Skill-based Clinical Assessment tool for Resuscitation (OSCAR) [16] in order to ensure comprehensive inclusion of skills. The OSACS assessment is based on 10 domains assessed on a 4 point anchored Likert scale (Fig. 1). The scale has descriptive explanations at each position. Total scores vary between 10 to 40, with higher scores indicating better performance. For the purpose of this study, four of the domains (leadership, medical record keeping, team working and handover) were not included, as they could not be directly assessed during the simulated scenarios. The lowest and highest achievable scores were therefore 6 and 24, respectively.

### Self-assessment

**Fig. 2:**
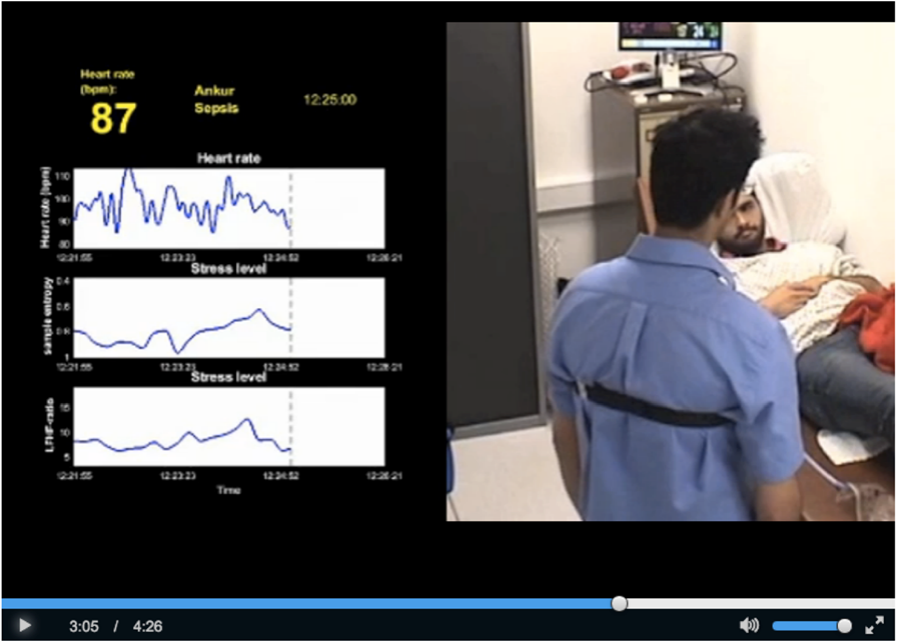
Screenshot of video replay with augmented biofeedback using HRV stress indices

Participants were approached individually up to 5 days following the simulation task. Following a brief tutorial regarding HRV stress indices and the OSACS assessment tool, videos augmented with real time display of HRV stress indices (Fig. 2) of their performance were replayed to them. For simplicity, only HR, SE and LF/HF HRV stress indices were displayed. Participants were then asked to assess their own performance for each scenario using the OSACS assessment tool.

Following the self-assessment, participants were asked to complete a post-study questionnaire to explore attitudes towards self-assessment and augmented video replay with biofeedback. Statements were as follows **1.** OSACS provides a valuable structure for self-assessment; **2.** Video replay was helpful in the process of self-assessment; **3.** Physiological signals were helpful in self-assessment; **4.** Overall, structured self-assessment is a valuable exercise; **5**. Structured self-assessment should be a regular part of medical training. Each question was scored on a 5-point Likert scale; 1. Strongly disagree, 2. Disagree, 3. Neither agree nor disagree, 4. Agree, 5. Strongly agree.

### Analysis

Data were not normally distributed and so non-parametric tests were used. The Kruskal-Wallis test was used to identify differences between three or more independent groups and the Mann-Whitney U test to identify differences between two independent groups. SPSS 23 (IBM corporation) was used in the statistical analysis. A *p*-value of < 0.05 was considered statistically significant.

## Results

### Demographics

There were 22 participants in total, of which 13 were male and 9 female. Participants were from a range of different specialties including Surgery, Anaeasthetics and Emergency Medicine. Sixty out of a possible 66 simulated scenarios were evaluated; due to clinical commitments some participants did not have enough time to complete all three clinical scenarios. Participants were categorized according to the grade of their current post; *Novice*: Year 1 Foundation Trainees, *Intermediate*: Year 2 Foundation Trainees to Year 2 Core Trainees, *Expert*: Specialist registrars and Consultants. Of the participants 7 were novices, 5 intermediates and 10 experts.

### OSACS scores

**Fig. 3:**
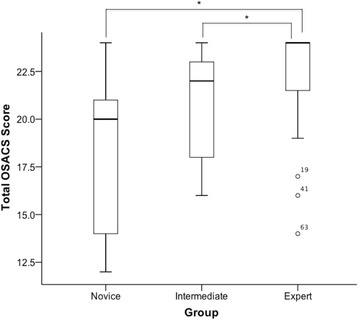
Box plot of experience vs median total OSACS scores (*p < 0.05)

Overall OSCAS scores for all clinical scenarios were initially analyzed. There were statistically significant differences in total OSACS scores across the three experience groups (Novice 20 [14–21], Intermediate 22 [18–23] and Expert 24 (21.8–24), *p* ≤ 0.001); Fig. 3. When comparing individual groups we found this difference to be significant between novices vs. experts (*p* < 0.001) and intermediates vs. experts (*p* = 0.037) but not between novices vs. intermediates (*p* = 0.11). Individual OSACS domains were also analyzed to establish the importance of each domain; Additional file 1: Table S1. Individual OSACS domains were also analyzed for each of the three clinical scenarios; Additional file 1: Table S1.

### HRV stress indices

At rest, there were no significant differences in any of the HRV stress indices between different experience groups; HR (*p* = 0.17), SE (*p* = 0.47), LFHF (*p* = 0.43), SDRR (*p* = 0.45), RMSSD (p = 0.17), PRR50 (*p* = 0.22).

When analyzing combined data for all clinical scenarios, not including resting periods, there was only a statistically significant difference in SDRR between the three different experience groups (Additional file 1: Table S1)**.** Further analysis demonstrated this statistically significant difference to be between novices and intermediates (*p* = 0.022) with a trend towards significance between novices and experts (0.08). There was also a trend to significance when comparing intermediates and experts for LFHF (*p* = 0.09). When analysing data for individual clinical scenarios we found no significant difference in any of the HRV indices between the different experience groups.

In order to account for the differences in resting HR and HRV indices for participants, differences between resting periods and simulated tasks were calculated (Additional file 2: Figure S1). We found a significantly larger decrease in RMSSD (− 0.12 (− 0.14 to − 0.09) vs − 0.05 (− 0.09 to 0.01), *p* = 0.024) and pRR50 (− 9.6 (− 11.3 to − 7.5) vs − 0.9 (− 6.5 to 1), p = 0.024) in the intermediate group as compared to the expert group respectively, indicating higher levels of stress during the simulation. Although not reaching statistical significance, the novice participants had a higher increase in HR, compared to expert participants (9.8 (6.6 to 15.2) vs 5.2 (− 1.3 to 10.5), *p* = 0.055).

There were no significant correlations between any of the HRV stress indices and total OSACS scores; HR (*R* = − 0.015, *p* = 0.92), SE (*R* = 0.009, *p* = 0.95), LFHF (*R* = − 0.009, p = 0.95), SDRR (*R* = 0.15, *p* = 0.28), RMSSD (*R* = 0.013, *p* = 0.93) and PRR50 (*R* = − 0.031, *p* = 0.83).

### Post study questionnaire

**Fig. 4:**
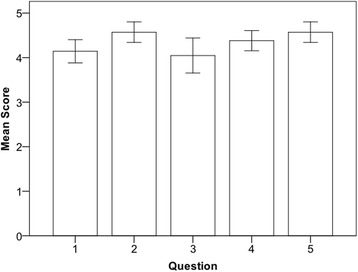
Bar chart displaying responses of participants to post study questionnaire. Question 1, OSACS provides a valuable structure for self-assessment; Question 2, Video replay was helpful in the process of self-assessment; Question 3, Physiological signals were helpful in self-assessment; Question 4, Overall structured self-assessment is a valuable exercise; Question 5, Structured self-assessment should be a regular part of medical training. Each question was scored on a 5-point Likert scale; 1. Strongly disagree, 2. Disagree, 3. Neither agree nor disagree, 4. Agree, 5. Strongly agree

The post study questionnaire was completed by 21 out of 22 participants. Overall, participant satisfaction was rated as high in the post study questionnaire. Each question was scored on a 5-point Likert scale; 1. Strongly disagree, 2. Disagree, 3. Neither agree nor disagree, 4. Agree, 5. Strongly agree. **Q1.** OSACS provides a valuable structure for self-assessment (mean 4.1 +/− 0.6); **Q2.** Video replay was helpful in the process of self-assessment (mean 4.6 +/− 0.5); **Q3.** Physiological signals were helpful in self-assessment (mean 4.0 +/− 0.9); **Q4.** Overall structured self-assessment is a valuable exercise (mean 4.4 +/− 0.5); **Q5.** Structured self-assessment should be a regular part of medical training (mean 4.6 +/− 0.5) (Fig. 4).

## Discussion

Work placed based assessments (WBA) are an essential and mandatory part of the Intercollegiate Surgical Curriculum Programme (ISCP) in the UK. Despite the emphasis placed on WBAs as a method of assessment and the time commitment required from both trainers and trainees, there is no evidence that their use leads to an improvement in performance [17]. Furthermore, the satisfaction with WBA amongst trainees and trainers remains low [18]. This highlights the need for continuous evaluation and refinement of assessment tools in surgical training in order to engage trainees the best.

Self-assessment is an important element of continued professional development and life long learning. Self-assessment may provide an efficient tool to enhance performance whilst reducing the time that is required for supervision and providing feedback. However, an important component of assessment is the availability of tools that capture performance in a valid and reliable manner.

In this study we have demonstrated the feasibility of self-assessment using an assessment tool alongside video replay augmented with stress biofeedback in a simulated setting. This can be used for both learning and self-assessment of surgical ward crisis management. Early recognition and postoperative management of complications play an important role in avoidance of preventable harm [19]. A report from the National Confidential Enquiry into Patient Outcome and Death described numerous deficiencies in the care of acutely ill ward patients [1]. The report also highlighted the need to better prepare trainees to manage acutely ill ward patients.

The accuracy of self assessment is significantly improved when trainees review video tapes of their performance [20] or compare their own performance against video taped benchmark [21]. Trainees can also improve their ability to self evaluate with practice and repetition [22]. Despite this, a key concern about self-assessment is that trainees with poor skills might not be aware of their deficiencies. In order to address this issue both an external assessor and trainee can use the OSACS assessment tool to assess performance. Scores can then be compared together during a debriefing and areas of disagreement explored in an open dialogue.

In this study the OSACS instrument was liked and valued by the participants who also agreed that structured self-assessment should form a regular part of medical training.

The primary aim of this study was to evaluate the feasibility and participant attitudes towards using video replay augmented with real time biofeedback. Although some stress can enhance performance, high levels of stress can be detrimental to various aspects of performance including decision making and communication [23]. Most trainees receive no formal training in stress management. However, if stress can be quantified in challenging clinical situations such as the management of surgical ward crisis then coping strategies may be better developed. Participants in our study agreed that the real time biofeedback given during video replay was beneficial in self-assessment.

OSACS scores did not correlate with stress, as measured by HRV stress indices. However, SDRR was significantly lower in the novice group of participants indicating higher stress levels. Variations in other HRV indices such as SE, LFHF and PRR50 did not reach statistical significance possibly secondary to a type II error. We have demonstrated that HRV stress indices show some promise in being able to differentiate between different levels of experience but more importantly provide useful physiological data for self-assessment at an individual level. Further work refining and evaluating this methodology is warranted. It would also be valuable for surgical training to quantify ‘optimal stress’ levels using HRV indices. Further work is also required to directly compare video replay with and without augmented biofeedback.

This study has a number of limitations. Firstly, evaluations were carried out in a simulated setting that does not reflect all the challenges faced in the clinical setting. Furthermore, a small number of participants were evaluated with unequal group sizes, possibly resulting in Type II error in some of our analysis.

## Conclusion

Self-assessment using an objective self-assessment tool alongside video replay augmented with stress biofeedback is feasible in a simulated setting and well liked by participants. Further work is required to explore the educational benefit of this approach.

## Additional files

Additional file 1: **Table S1.** OSACS scores, time to diagnosis and individual HRV stress indices for different experience groups and individual scenarios. (DOCX 22 kb)

Additional file 2: **Figure S1.** Difference between simulation and resting HRV indices between different experience groups. (PNG 176 kb)

## Abbreviations

ADC: Analog-to-digital converter
ANS: Autonomic nervous system
DVT: Deep vein thrombosis
ECG: Electrocardiogram
HR: Average heart rate
HRV: Heart rate variability
ISCP: Intercollegiate surgical curriculum programme
LF/HF: Low frequency/high frequency ratio
NOTECHS: Non-technical skills
OSACS: Objective structured assessment of acute care skills
OSCAR: Observational skill-based clinical assessment tool for resuscitation
PE: Pulmonary embolism
PRR50: Percentage of RR interval pairs with difference > 50 ms
RMSSD: Root mean squared of successive differences
SDRR: Standard deviation of RR Intervals
SE: Sample entropy
WBA: Work placed based assessment

## References

[1] National Confidential Enquiry into Patient Outcome and Death (2005). An acute problem?.

[2] Arora S, Hull L, Fitzpatrick M, Sevdalis N, Birnbach DJ (2015). Crisis management on surgical wards: a simulation-based approach to enhancing technical, teamwork, and patient interaction skills. Ann Surg.

[3] Rizan C, Ansell J, Tilston TW, Warren N, Torkington J (2015). Are general surgeons able to accurately self-assess their level of technical skills?. Ann R Coll Surg Engl.

[4] Spencer JA, Jordan RK (1999). Learner centred approaches in medical education. BMJ.

[5] Hassan I, Weyers P, Maschuw K, Dick B, Gerdes B, Rothmund M (2006). Negative stress-coping strategies among novices in surgery correlate with poor virtual laparoscopic performance. Br J Surg.

[6] Yerkes R, Dodson J (1908). The relation of strength of stimulus to rapidity of habit formation. J Comp Neurol Psychol.

[7] Lazarus RS (1985). The psychology of stress and coping. Issues Ment Health Nurs.

[8] Kumar M, Weippert M, Vilbrandt R, Kreuzfeld S, Stoll R (2007). Fuzzy evaluation of heart rate signals for mental stress assessment. IEEE trans fuzzy Syst.

[9] Salahuddin L, Cho J, Jeong MG, Kim D (2007). Ultra short term analysis of heart rate variability for monitoring mental stress in mobile settings. Conf Proc IEEE Eng Med Biol Soc.

[10] Bozhokin SV, Shchenkova IM (2008). Analysis of the heart rate variability using stress tests. Hum Physiol.

[11] Schubert C, Lambertz M, Nelesen RA, Bardwell W, Choi J-B, Dimsdale JE (2009). Effects of stress on heart rate complexity—a comparison between short-term and chronic stress. Biol Psychol.

[12] Gutierrez-Osuna R, Choi J. Using heart rate monitors to detect mental stress. 2009 sixth international workshop on wearable & implantable body sensor networks conference (BSN 2009)(BSN), Berkeley, CA. 2009. pp. 219–23.

[13] Chanwimalueang T, Von Rosenberg W, Mandic DP (2015). Enabling R-peak detection in wearable ECG: combining matched filtering and Hilbert transform. Proc IEEE Int conf digit signal process.

[14] Gagne R (1985). The conditions of learning (4th ed.).

[15] Sevdalis N, Davis R, Koutantji M, Undre S, Darzi A, Vincent CA (2008). Reliability of a revised NOTECHS scale for use in surgical teams. Am J Surg.

[16] Walker S, Brett S, McKay A, Lambden S, Vincent C, Sevdalis N (2011). Observational skill-based clinical assessment tool for resuscitation (OSCAR): development and validation. Resuscitation.

[17] Miller A, Archer J (2010). Impact of workplace based assessment on doctors’ education and performance: a systematic review. BMJ.

[18] Pereira EAC, Dean BJF (2009). British surgeons’ experiences of mandatory online workplace-based assessment. J R Soc Med.

[19] Sheetz KH, Waits SA, Krell RW, Campbell DA, Englesbe MJ, Ghaferi AA (2013). Improving mortality following emergent surgery in older patients requires focus on complication rescue. Ann Surg.

[20] Ward M, MacRae H, Schlachta C, Mamazza J, Poulin E, Reznick R, et al. Resident self-assessment of operative performance. Am J Surg 2003 185(6):521–4. 10.1016/s0002-9610(03)00069-2 12781878

[21] Martin D, Regehr G, Hodges B, McNaughton N (1998). Using videotaped benchmarks to improve the self-assessment ability of family practice residents. Acad Med.

[22] MacDonald J, Williams RG, Rogers DA (2003). Self-assessment in simulation-based surgical skills training. Am J Surg.

[23] Wetzel CM, Kneebone RL, Woloshynowych M, Nestel D, Moorthy K, Kidd J (2006). The effects of stress on surgical performance. Am J Surg.

