# Supplementary material for: Self-assessment of surgical ward crisis management using video replay augmented with stress biofeedback
Source: Patient Saf Surg. 2018 Apr 19;12:6. doi: 10.1186/s13037-018-0153-5 (PMC5907372; doi:10.1186/s13037-018-0153-5)
Supplement: Supplementary file 1 — Table S1. OSACS scores, time to diagnosis and individual HRV stress indices for different experience groups and individual scenarios. (DOCX 22 kb) [file 13037_2018_153_MOESM1_ESM.docx]

|  | **Case 1 (PE)** | | | | **Case 2 (Bleeding)** | | | | **Case 3 (Sepsis)** | | | | **All** | | | |
| --- | --- | --- | --- | --- | --- | --- | --- | --- | --- | --- | --- | --- | --- | --- | --- | --- |
| **Domain** | **Novice** | **Intermediate** | **Expert** | **p** | **Novice** | **Intermediate** | **Expert** | **p** | **Novice** | **Intermediate** | **Expert** | **p** | **Novice** | **Intermediate** | **Expert** | **p** |
| 1 | 3 (3-4) | 3 (3-3.5) | 4 (4-4) | 0.10 | 3(3-3.5) | 4(3.5-4) | 4(4-4) | 0.20 | 3(3-3.5) | 4(3.5-4) | 4(4-4) | 0.3 | 3(3-4) | 4(3-4) | 4(4-4) | *0.012 |
| 2 | 4 (3-4) | 4 (3.5-4) | 4 (4-4) | 0.89 | 3(3-3.5) | 4(4-4) | 4(3-4) | 0.10 | 3(2.5-4) | 4(3.5-4) | 4(3.25-4) | 0.45 | 3(3-4) | 4(4-4) | 4(3-4) | 0.11 |
| 3 | 4 (3.5-4) | 3 (2.5-3.5) | 4 (4-4) | 0.25 | 3(2.5-3) | 4(3-4) | 4(3-4) | *0.026 | 3(2-3.5) | 4(3.5-4) | 4(4-4) | 0.09 | 3(3-4) | 4(3-4) | 4(3.75-4) | *0.01 |
| 4 | 4 (3-4) | 3 (3-3.5) | 4 (4-4) | 0.17 | 3(2-3) | 3(2.5-3.5) | 4(4-4) | *0.021 | 2(2-3) | 3(2.5-3.5) | 4(4-4) | *0.003 | 3(2-3) | 3(3-4) | 4(4-4) | *<0.001 |
| 5 | 3 (3-3.5) | 3 (3-3.5) | 4 (4-4) | 0.13 | 3(2.5-3.5) | 3(3-3.5) | 4(4-4) | 0.18 | 3(2-3.5) | 3(2.5-3.5) | 4(3.24-4) | 0.163 | 3(3-4) | 3(3-4) | 4(3.75-4) | *0.004 |
| 6 | 3 (3-3) | 3 (3-3.5) | 4 (4-4) | *0.011 | 2(2-3) | 3(3-3.5) | 4(4-4) | *0.015 | 2(1.5-3.5) | 3(3-3.5) | 4(4-4) | *0.039 | 3(2-3) | 3(3-4) | 4(4-4) | *<0.001 |
| **Total** | 20 (19.5-22) | 18 (18-21) | 24 (23-24) | 0.13 | 16(14-20) | 22(19.5-22.5) | 23(21-24) | *0.014 | 14(13.5-21) | 22(19-22.5) | 24(22-24) | *0.03 | 20(14-21) | 22(18-23) | 24(21.75-24) | *<0.001 |
| **Time to diagnosis** | 1.2 (0.15-4) | 0 (0-0.35) | 1.13 (0-2.03) | 0.35 | 3(2.40-5.05) | 0.3(0.15-0.45) | 1.48(1.02-2.3) | *0.031 | 9.07(2.44-9.04) | 1.3(0.45-2.15) | 2(0.31-2.23) | *0.04 | 3(2-6) | 0(0-1.08) | 1.43(0.22-2.19) | *0.001 |
| **HRV stress indices** | | | | | | | | | | | | | | | | |
| ***HR*** | 92.3 (86.2-120.5) | 84.97(79.4-87.6) | 89.8(81.1-96.1) | 0.45 | 98.8(83-113.5) | 90.5(76.5-93.7) | 87.9(82.9-97.8) | 0.47 | 93.4(82.7-110.5) | 94.5(77.3-95.7) | 91.5(83.4-95.4) | 0.84 | 93.4(81.4-117.4) | 87.8(68.5-94.8) | 91.3(82.3-97.4) | 0.18 |
| ***SE*** | 0.83 (0.73-0.89) | 0.83(0.72-0.94) | 0.73(0.72-0.83) | 0.78 | 0.86(0.76-0.93) | 0.77(0.72-1) | 0.87(0.71-0.89) | 0.97 | 0.88(0.77-0.97) | 0.7(0.67-0.98) | 0.8(0.74-0.86) | 0.88 | 0.85(0.72-0.94) | 0.76(0.67-0.98) | 0.78(0.72-0.89) | 0.85 |
| ***LFHF*** | 6.98 (5.69-7.94) | 4.15(3.16-6.35) | 8.24(6.42-8.69) | 0.53 | 7.64(4.77-9.95) | 3.4(2.69-6.31) | 8.72(4.79-11.6) | 0.34 | 3.90(2.74-6.11) | 3.37(2.89-6.90) | 7.57(5.7-9.9) | 0.41 | 6(3.9-8.2) | 3.39(2.97-8.17) | 7.83(4.96-9.64) | 0.14 |
| ***SDRR*** | 0.04 (0.03-0.05) | 0.06(0.055-0.06) | 0.055(0.048-0.06) | 0.15 | 0.04(0.04-0.06) | 0.08(0.06-0.08) | 0.05(0.04-0.06) | 0.32 | 0.05(0.04-0.06) | 0.07(0.055-0.075) | 0.06(0.04-0.07) | 0.29 | 0.04(0.03-0.06) | 0.06(0.05-0.08) | 0.06(0.04-0.06) | *0.04 |
| ***RMSSD*** | 0.66 (0.5-0.71) | 0.715 (0.69-0.78) | 0.68(0.62-0.75) | 0.45 | 0.62(0.53-0.74) | 0.68(0.65-0.825) | 0.69(0.62-0.74) | 0.50 | 0.65(0.55-0.74) | 0.64(0.63-0.83) | 0.67(0.64-0.72) | 0.84 | 0.65(0.51-0.74) | 0.7(0.64-0.9) | 0.67(0.62-0.74) | 0.18 |
| ***pRR50*** | 4.20 (0.5-5.23) | 9.29(5.5-15.81) | 5.71(3.04-7.46) | 0.28 | 9.1(1.35-9.77) | 6.41(4.14-23.15) | 6.24(0.79-10.12) | 0.80 | 7.63(0.72-11.01) | 3.81(2.18-24.02) | 5.85(0.47-12.43) | 0.91 | 4.47(0.82-9.68) | 6.77(2.36-24.5) | 6.10(1.37-10.20) | 0.51 |
