# Supplementary figures and images for: Self-assessment of surgical ward crisis management using video replay augmented with stress biofeedback
Source: Patient Saf Surg. 2018 Apr 19;12:6. doi: 10.1186/s13037-018-0153-5 (PMC5907372; doi:10.1186/s13037-018-0153-5)

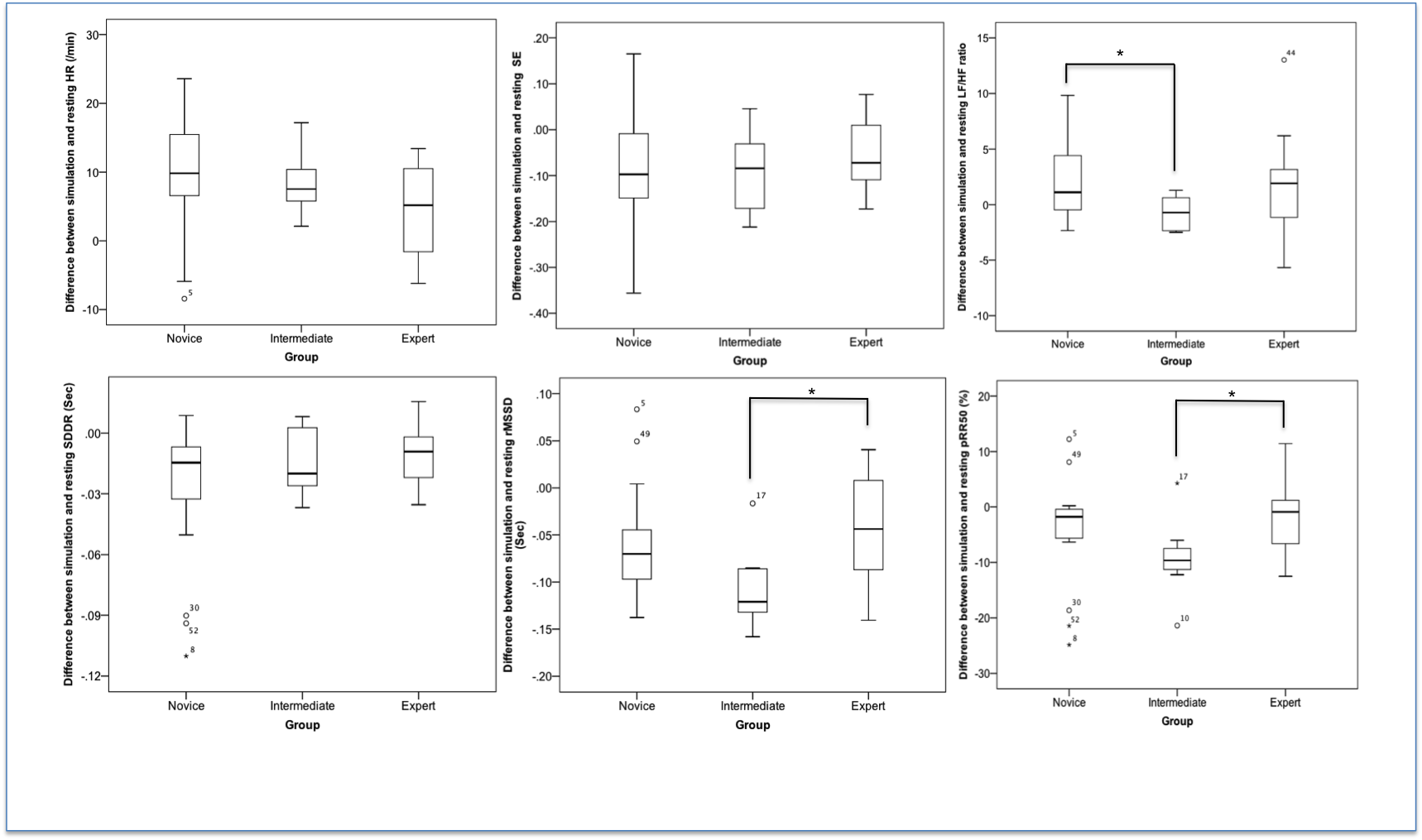

Supplement: Supplementary file 2 — Figure S1. Difference between simulation and resting HRV indices between different experience groups. (PNG 176 kb) [file 13037_2018_153_MOESM2_ESM.png]
